# Single nucleotide vitamin D receptor polymorphisms (FokI, BsmI, ApaI, and TaqI) in the pathogenesis of prematurity complications

**DOI:** 10.1038/s41598-020-78125-4

**Published:** 2020-12-03

**Authors:** Katarzyna Kosik, Dawid Szpecht, Salwan R. Al-Saad, Lukasz M. Karbowski, Grażyna Kurzawińska, Marta Szymankiewicz, Krzysztof Drews, Hubert Wolski, Agnieszka Seremak-Mrozikiewicz

**Affiliations:** 1 Chair and Department of Neonatology, Poznan University of Medical Sciences, Poznan, Poland; 2 Poznan University of Medical Sciences, Poznan, Poland; 3 Department of Perinatology and Women’s Diseases, Poznan University of Medical Sciences, Poznan, Poland

**Keywords:** Diseases, Molecular medicine, Pathogenesis, Risk factors

## Abstract

The vitamin D receptor (VDR), coded by the VDR gene, plays a pivotal role in executing cellular functions when bound by the active form of vitamin D. Gene polymorphisms in this receptor have been increasingly associated with a heightened state of vulnerability to certain diseases. However, limited data is available concerning the role of VDR gene polymorphisms in preterm infant complications. In 114 premature infants (< 32 weeks gestation) we analyze four single nucleotide VDR polymorphisms (rs2228570 (FokI), rs1544410 (BsmI), rs797532 (ApaI), rs731236 (TaqI)) for their association with respiratory distress syndrome (RDS), intraventricular hemorrhage (IVH), bronchopulmonary dysplasia (BPD), necrotizing enterocolitis (NEC) and retinopathy of prematurity (ROP). The results show that BPD was almost four times more likely in infants with the genotype CC of ApaI (rs7975232) (OR 3.845; p = 0.038). While both BPD and NEC were 2.1 times more likely to occur in preterm infants with the allele C of ApaI (rs7975232) (respectively: OR 2.111 and OR 2.129, p < 0.05). The ApaI VDR polymorphism appears to influence incidence of BPD and NEC in preterm infants. Considering VDR polymorphisms in future genetic investigations, in preterm complications, may prove clinically relevant.

## Introduction

Vitamin D activity is regulated by the vitamin D receptor (VDR), also known as the calcitriol receptor or Nr1I1. It is a nuclear transcription factor that, when activated by a ligand, modulates gene expression^1^. VDR is expressed extensively in immune system cells, thymus, placenta, alveolar type-II cells, osteoblasts, chondrocytes, and skin epithelium^2,3^. In contrast to the later, erythrocytes, mature striated muscle cells, and highly differentiated neural cells of the cerebral cortex present considerably less vitamin D receptors^4^.

In the human body, most of the utilized vitamin D is sourced by endogenous cutaneous production. Pre-vitamin D is provided by photoisomerization from 7-dehydrocholesterol (ergosterol) due to ultraviolet radiation (UV-B). Then, it is transformed to cholecalciferol, and after hydroxylation in the liver and kidneys—to calcitriol. This active hormonal form binds to the vitamin D binding protein (VDBP) in the blood and reaches the target cell. Once it enters the cell, it attaches to VDR in the cytoplasm and constructs a combined complex. This fusion enters the nucleus, where it heterodimerizes with retinoic acid X receptor and enhances vitamin D-dependent gene transcription^5^. The resulting interactions trigger the tissue-specific responses and their molecularly correlated cascading effects.

As with many types of receptors, the genes responsible for coding the VDR present with subtle allelic variants located on chromosome 12^6^. To date, the most explored VDR polymorphisms are rs2228570 (FokI), rs1544410 (BsmI), rs797532 (ApaI), rs731236 (TaqI)^7^. TaqI and FokI consist of a single nucleotide change—A to G and G to A in exons 9 and 2, respectively. Whilst BsmI and ApaI are located in intron 8 and result in a change from G to A and A to C, respectively. Possible associations are being explored between VDR gene variation and the development of different types of cancers (ApaI polymorphism in the VDR gene may prove to be a good marker for the use of individualized chemotherapy in the treatment of non-small cell lung carcinoma)^8^, autoimmune diseases (risk of developing multiple sclerosis is still being investigated)^9^, diabetes^10,11^, asthma^12^, cardiovascular diseases^13^, osteoporosis^14^, and susceptibility to infection^15^.

The following work is centred to investigate VDR polymorphisms (FokI, BsmI, ApaI, TaqI) and their association with risk for neonatal complications such as respiratory distress syndrome, intraventricular hemorrhage, bronchopulmonary dysplasia, necrotizing enterocolitis and retinopathy of prematurity.

## Material and methods

### Study population

The study comprises a population of 114 premature infants born within 22 + 6 to 32 + 0 weeks of gestation, between the years 2014 and 2019. All infants were hospitalized at the Department of Neonatology (III Level Hospital) located in the Clinical Hospital of Gynecology and Obstetrics at Poznan University of Medical Sciences in Poznan, Poland. Homogenic consistency was maintained as a result of all infants being of Caucasian origin. Excluded were neonates born after 32 + 0 weeks of pregnancy, multiple pregnancy births, infants with chromosomal abnormalities, TORCH infections (rubella, herpes, cytomegalovirus, toxoplasmosis, etc.) and infants without antenatal steroid therapy.

### Clinical features

Clinical factors were observed for association with VDR gene polymorphism and its influence on neonatal development and preterm complications. The clinical features include: gender, gestational age (GA; weeks), birth weight (BW, grams), mode of delivery (vaginal birth vs cesarean section), APGAR score in 1st and 5th minute, and pH and blood base excess (BE) in cord blood. The five preterm complications evaluated were respiratory distress syndrome (RDS), intraventricular hemorrhage (IVH), bronchopulmonary dysplasia (BPD), necrotizing enterocolitis (NEC) and retinopathy of prematurity (ROP). Infants who were delivered outside third-level hospitals were also analyzed for developing complications of preterm birth.

### Diagnosis

#### RDS

The diagnosis of RDS is based on a clinical picture of a preterm infant with the onset of progressive respiratory failure shortly after birth^16^. This is manifested by breathing disorders and an increase in the oxygen requirement, in conjunction with a characteristic chest radiograph or ultrasound (4–15 MHz transducer, MyLabEight, Esaote). Criteria for the classification of RDS was obtained from European Consensus Guidelines on the Management of Respiratory Distress Syndrome^17^.

#### IVH

Intraventricular hemorrhage was diagnosed with the use of cranial ultrasound (10 MHz transducer, Prosundα7 Premier or Aloka; 4–15 MHz transducer, MyLabEight, Esaote). Routine cranial ultrasound screenings were performed on the 1st, 3rd and 7th days post birth in accordance with the local hospital standards. Classification of IVH was obtained using criteria based on the Papile IVH Severity Scale^18^.

#### BPD

Bronchopulmonary dysplasia was defined as the need for at least one form of oxygen supplementation either at 28 postnatal days or 36 weeks postmenstrual age (PMA)^19^. Notably, this definition does not take into account the severity of respiratory disease or extent of prematurity^20–22^.

#### NEC

Necrotizing enterocolitis is characterized by ischemic necrosis of the intestinal mucosa, which is associated with severe inflammation, invasion of enteric gas forming organisms, and dissection of gas into the bowel wall and portal venous system^23^. Severity of NEC can vary in degree in preterm neonates and can manifest in variable stages. Diagnosis of NEC was based of the Modified Bell’s clinical criteria assessment of NEC stages using an abdomen ultrasound (5–8 and 13 MHz transducer, Prosundα7 Premier, Aloka or 4–15 MHz transducer, MyLabEight, Esaote) and an X-Ray of the abdomen^24^. Within the Modified Bell’s clinical criteria, stages were divided into three specific components for the most accurate recognition and diagnosis of NEC^25^.

#### ROP

Retinopathy of prematurity is classified as a developmental vascular proliferative disorder that occurs in the retina of preterm infants with incomplete retinal vascularization. The screening examination followed the guidelines of Polish Ophthalmological Society for ROP screening (≤ 33 weeks of postmenstrual age, ≤ 1800 g of birth weight or if they were determined to be at high risk by a neonatologist)^26^. Patients were examined with binocular indirect ophthalmoscopy by a team of three trained paediatric ophthalmologists specializing in ROP. The initial fundus examination was performed 4 weeks postnatally, with further follow-up every 7–10 days until full vascularization of the retina, stabilization of the changes or the necessity for treatment. Findings were classified according to the Revisited International Classification of Retinopathy of Prematurity (ICROP)^27,28^.

### Studied polymorphisms

The criteria for selections of candidate genes in the present study were their potential involvement in the pathogenesis of RDS, IVH, NEC, BPD or ROP. We studied 4 single nucleotide polymorphisms of vitamin D receptor: rs2228570 (FokI), rs1544410 (BsmI), rs797532 (ApaI), rs731236 (TaqI).

A sample of blood was taken following birth, after admission to the intensive care unit. Genomic DNA was extracted from blood leukocytes using QIAamp DNA Blood Mini Kit (QIAGEN Inc; Germany). Genotyping was performed using polymerase chain reaction/restriction fragment length polymorphism (PCR/RFLP) procedures. For detection of rs2228570 mutation PCR was amplified with starters: 5′-AGCTGGCCCTGGCACTGACTCTGCTCT-3′, 5′-ATGGAAACACCTTGCTTCTTCTCCCTC-3′ (PCR product 267 bp long) and hydrolyzed at 37 °C with FokI restriction enzyme (Eurx, Poland). The following genotypes were obtained**:** CC (FF) 267 bp; CT (Ff) 267, 197, 70 bp and TT (ff) 197, 70 bp. For detection of the rs1544410 mutation, PCR was amplified with starters: 5′-CAACCAAGACTACAAGTACCGCGTCAGTGA-3′, 5′-AACCAGCGGGAAGAGGTCAAGGG-3′ (PCR product 821 bp long) and hydrolyzed at 37 °C with Mva12691 (BsmI) restriction enzyme (Thermo Scientific, USA). The following genotypes were obtained: GG (bb) 646, 175 bp; GA (Bb) 821, 646,175 bp; AA (BB) 821 bp. The rs7975232 and rs731236 polymorphic sites of the *VDR* gene were detected using starters: 5′-CAGAGCATGGACAGGGAGCAA-3′, 5′-GCAACTCCTCATGGCTGAGGTCTC-3′. PCR product (745 bp long) was hydrolyzed with ApaI restriction enzyme at 25 °C overnight (Eurx, Poland) and found the following genotypes: AA (AA) 745 bp; AC (Aa) 745, 528, 217 bp and CC (aa) 528 bp, 217 bp. For detection of the rs731236 polymorphism, 745 bp PCR product was hydrolyzed overnight at 65 °C with TaqI restriction enzyme (Eurx, Poland). TaqI has two binding sites on the PCR product and produces a following fragments: TT (TT) 494, 251 bp; TC (Tt) 494, 293, 251, 201 bp and CC (tt) 293, 251, 201 bp. DNA fragments obtained after respective restrictive enzyme digestion and the DNA size marker were electrophoresed on a 2.5% agarose gel with Midori Green Advanced DNA Stain (Nippon Genetics, Europe GmbH).

Description of the studied polymorphism genes is presented Table 1.

**Table 1:** Primer sequences, location of the primer pairs, and fragment sizes of the vitamin D receptor gene polymorphisms.

VDR polymorphism	Location	Primers	Enzyme	Product
rs2228570	Exon 2 (chr12:47879112)	5′-AGCTGGCCCTGGCACTGACTCTGCTCT-3’ 5′-ATGGAAACACCTTGCTTCTTCTCCCTC-3’	FokI	C (F): 267 bpT (f): 197 bp, 70 bp
rs1544410	Intron 8 (chr12:47846052)	5′-CAACCAAGACTACAAGTACCGCGTCAGTGA-3’ 5′-AACCAGCGGGAAGAGGTCAAGGG-3’	BsmI	G (b): 646 bp, 175 bp A (B): 821 bp
rs7975232	Intron 8 (chr12:47845054)	5′-CAGAGCATGGACAGGGAGCAA-3' 5′-GCAACTCCTCATGGCTGAGGTCTC-3’	ApaI	A (A): 745 bp C (a): 528 bp, 217 bp
rs731236	Exon 9 (chr12:47844974)		TaqI	T (T): 494 bp, 251 bp C (t): 293 bp, 251 bp, 201 bp

VDR polymorphisms: FokI (rs2228570); TaqI (rs731236); ApaI (rs7975232); BsmI (rs1544410); chr12—chromosome 12; Allels: C, T, G, A.

### Ethics statement

All procedures performed in studies involving human participants were in accordance with the ethical standards of the institutional and/or national research committee and with the 1964 Helsinki declaration and its later amendments or comparable ethical standards. The study was approved by the Bioethics Committee of Poznan University of Medical Sciences (no. 66/14 and 799/16). Informed consent was obtained from all parents.

### Statistical analysis

Statistical analysis was performed using CytelStudio version 11.1.0 (CytelStudio Software Corporation, Cambridge, Massachusetts, United States) and Statistica version 10 (Stat Soft, Inc., Tulsa, Oklahoma, United States). A *p* value of less than 0.05 indicates statistical significance. Categorical variables were presented as a percentage. Non-normally distributed continuous variables were tested by the Shapiro–Wilk test and showed as median and ranges. To evaluate the association between prematurity complications and categorical variables the following test were performed: the Fisher exact probability test, the Chi-square test, Fisher Freeman Halton, and Chi-squared test with Yates correction. U Mann–Whitney test was used to analyze differences in non-normally distributed continuous variables.

## Results

### Complications of prematurity

#### IVH

Table 2 presents demographic and clinical features of enrolled patients. Of the 114 infants, 55 (48.2%) developed IVH. The infants were diagnosed according to stages of IVH; 20 (36.4%) newborns were diagnosed with IVH stage I, 25 (45.5%) with IVH stage II, 10 (18.2%) with stage III and no neonates with stage IV. In our study population, male infants were significantly more likely to develop IVH stage II/III (26 (74.3%) male vs 9 (25.7%) female, p = 0.0251). Furthermore, the frequency of IVH stage II–IV was inversely correlated to gestational age [prevalence was higher in children born before 29 weeks of gestation as compared to those after 29 weeks of gestation (88.6% vs 11.4% respectively, p < 0.001)]. Incidence of IVH was also higher in newborns with birth weight less than 1000 g (77.1% vs 22.9%, p < 0.001), with lower Apgar scores in first (5.48 vs 3.6, p = 0.0001) and fifth minute of life (7.4 vs. 6.37, p = 0.001), as well as with intrauterine infection (85.7% vs 14.3%, p < 0.001). 9 of the 55 (11.4%) neonates with IVH died during hospitalization (p = 0.004).

**Table 2:** Demographic and clinical characteristics of enrolled infants.

	Complications														
	RDS			IVH (II–IV)			BPD			NEC			ROP		
	Yes	No	p value	Yes	No	p value	Yes	No	p value	Yes	No	p value	Yes	No	p value
**Gender**															
Male	63	4	0.7597^a^	26	41	0.0251^e^	23	44	0.8393^e^	16	51	0.8402^e^	27	40	0.8098^e^
Female	44	3		9	38		17	30		12	35		20	27	
**Gestational age (week)**															
< 29	65	2	0.0953^a^	31	36	< 0.00001^a^	34	33	0.0001^a^	21	46	0.0446^e^	37	30	0.0003^e^
≥ 29	42	5		4	43		6	41		7	40		10	37	
**Birth weight (g)**															
< 750	19	1	0.1129^b^	11	9	0.0001^e^	13	7	< 0.00001^e^	6	14	0.1134^e^	14	6	0.0033^e^
750–1000	34	0		16	18		18	16		12	22		16	18	
> 1000	54	6		8	52		9	51		10	50		17	43	
**Apgar score**															
1st minute	5 (1–10)	6 (1–10)	0.5796^c^	4 (1–7)	6 (1–10)	0.0001^c^	4 (1–7)	6 (1–10)	0.0048^c^	5 (1–9)	6 (1–10)	0.4195^c^	4 (1–6)	6 (1–10)	0.028^c^
5th minute	7 (1–10)	7 (5–10)	0.9321^c^	7 (1–10)	7 (2–10)	0.001^c^	7 (1–10)	7 (4–10)	0.0029^c^	7 (5–10)	7 (1–10)	0.8434^c^	7 (4–9)	7 (1–10)	0.0951^c^
**Mode of delivery**															
Vaginal	46	5	0.283^a^	19	32	0.1723^e^	20	31	0.406^e^	16	35	0.1285^e^	22	29	0.7095^e^
Cesarean section	61	2		16	47		20	43		12	51		25	38	
**pH**	7.32 (6.91–7.53)	7.29 (7.07–7.51)	0.5693^c^	7.29 (6.98–7.51)	7.33 (6.91–7.53)	0.2098^c^	7.34 (6.91–7.51)	7.31 (7.02–7.53)	0.5668^c^	7.37 (7.16–7.53)	7.31 (6.91–7.51)	0.0304^c^	7.31 (6.91–7.4)	7.32 (7–7.53)	0.31^c^
**BE**	− 2.6 (− 21.9 to 10.6)	− 3.5 (− 9.4 to − 0.4)	0.2768^c^	− 2.9 (− 16.9 to 0.8)	− 2.8 (− 21.9 to 10.6)	0.4007^c^	− 2.35 (− 21.9 to 2.3)	− 3 (− 14.7 to 10.6)	0.5118^c^	− 2.02 (− 9.9 to 2.8)	− 3 (− 21.9 to 10.6)	0.1274^c^	− 3.03 (− 21.9 to 0.7)	− 2.85 (− 14.7 to 10.6)	0.2174^c^
**Intrauterine infection**															
Yes	65	5	0.8716^a^	30	40	0.0008^a^	29	41	0.0736^e^	21	49	0.0889^e^	32	38	0.2197^e^
No	42	2		5	39		11	33		7	37		15	29	
**In-born**	98	6	0.4837^d^	31	73	0.7577^a^	38	66	0.484^a^	26	78	0.9731^a^	44	60	0.6753^a^
**Out-born**	9	1		4	6		2	8		2	8		3	7	
**Deaths**															
Yes	12	1	0.7143^a^	9	4	0.004^a^	0	13	0.0038^d^	6	7	0.1143^a^	0	13	0.0007^d^
No	95	6		26	75		40	61		22	79		47	54	

Dell Statistica(data analysis software system), version 10. software.dell.com.

*BPD* bronchopulmonary dysplasia, *IVH* intraventricular hemorrhage, *NEC* necrotizing enterocolitis, *RDS* respiratory distress syndrome, *ROP* retinopathy of prematurity.

^a^Chi-square test with Yate's correction.

^b^Fisher Freeman Halton test.

^c^Mann Whitney test; ^d^Fisher's exact test; ^e^ Chi-square test.

#### RDS

RDS developed in 107/114 (93.9%) infants. No significant differences were observed in the clinical characteristics between the RDS and non-RDS infants, as seen in Table 2.

#### BPD

Furthermore, forty of the 114 infants (35.1%) suffered from BPD. Frequency of BPD was higher in children born before 29 weeks of gestation (85.0% vs 15.0%, p = 0.001). BPD incidence was also more pronounced in newborns with birth weight less than 1000 g (77.5% vs 22.5%, p < 0.001), or had lower Apgar scores in first (5.38 vs 4.03, p = 0.0048) and fifth minute of life (7.4 vs. 6.3; p = 0.0029).

#### NEC

Almost a quarter of the included infants developed NEC (28/114; 24.6%). The incidence of NEC was observed to be higher in children born before 29 weeks of gestation (75.0% vs 25.0%, p = 0.0446).

#### ROP

In the 47/114 (41.2%) infants who developed ROP, incidence was significantly higher in those with earlier gestation (78.7% in infants < 29 weeks of gestation as compared to 21.3% in those older, p < 0.001). Higher rate of ROP was also recorded in infants with birth weight less than 1000 g (63.8% vs 36.2%, p = 0.0033), and with lower Apgar score in the first minute (5.01 vs 4.11, p = 0.028).

#### VDR polymorphisms and complications of prematurity

Analysis showed higher prevalence of BPD in premature infants with the genotype CC ApaI (rs7975232) gene polymorphism [OR 3.845 (1.065–14.82); p = 0.038]. Furthermore, the study revealed that BPD and NEC were approximately two times more likely to occur in infants with the allele C of ApaI (rs7975232) [respectively: OR 2.111 (1.075–4.242), p = 0.028; and OR 2.129 (1.169–3.912), p < 0.05]. No other significant associations were found with the rest of the polymorphisms. Genotype and allele distribution in infants with complications of prematurity is presented in Table 3.

**Table 3:** Genotype and allele distribution of infants with or without RDS, BPD, IVH, NEC or ROP.

Polymorphism	Complications																			
	RDS				IVH (II–IV)				NEC				BPD				ROP			
	Yes	No	p value	OR	Yes	No	p value	OR	Yes	No	p value	OR	Yes	No	p value	OR	Yes	No	p value	OR
**VDR FokI (rs2228570)**																				
Genotype																				
CC	33	3		References	10	26		References	6	30		References	12	24		References	15	21		References
CT	20	2	1	0.909 (0.095—11.78)	18	38	0.838	1.232 (0.451—3.489)	16	40	0.290	2 (0.640—6.963)	18	38	1	0.947 (0.357—2.57)	22	34	0.989	0.906 (0.356—2.328)
TT	54	2	0.596	2.455 (0.264—30.51)	7	15	0.967	1.213 (0.318—4.43)	6	16	0.521	1.875 (0.42—8.242)	10	12	0.518	1.667 (0.487—5.65)	10	12	0.99	1.167 (0.349—3.861)
Allele																				
C	86	8		References	38	90		References	28	100		References	42	86		References	52	76		References
T	128	6	0.333	1.984 (0.579—7.179)	32	68	0.816	1.115 (0.607—2.038)	28	72	0.362	1.389 (0.724—2.66)	38	62	0.499	1.255 (0.699—2.248)	42	58	0.941	1.058 (0.601—1.862)
**VDR TaqI (rs731236)**																				
Genotype																				
CC	8	0		References	2	6		References	1	7		References	3	5		References	4	4		References
TC	57	5	1	0 (0—9.266)	17	45	1	1.133 (0.179—12.52)	12	50	1.000	1.68 (0.184—81.94)	16	46	0.747	0.579 (0.1—4.19)	22	40	0.667	0.55 (0.093—3.289)
TT	42	2	1	0 (0—30.55)	16	28	0.853	1.714 (0.262—19.14)	15	29	0.435	3.621 (0.394—173.9)	21	23	0.889	1.522 (0.257—10.93)	21	23	1	0.913 (0.149—5.583)
Allele																				
C	73	5		References	21	57		References	14	64		References	22	56		References	30	48		References
T	141	9	1	1.073 (0.272—3.72)	49	101	0.461	1.317 (0.693—2.551)	42	108	0.127	1.778 (0.868—3.801)	58	92	0.153	1.605 (0.857—3.06)	64	86	0.64	1.191 (0.657—2.173)
**VDR ApaI (rs7975232)**																				
Genotype																				
AA	21	2		References	6	17		References	3	20		References	6	17		References	9	14		References
AC	53	5	1	1.01 (0.089—6.778)	16	42	1	1.079 (0.327 -	12	46	0.649	1.739 (0.403—10.57)	15	43	1	0.988 (0.297—3.644)	20	38	0.883	0.819 (0.273—2.552)
CC	33	0	0.329	-	13	20	0.457	1.842 (0.507—7.195)	13	20	0.059	4.333 (0.954—26.65)	19	14	0.038	3.845 (1.065—14.82)	18	15	0.388	1.867 (0.558—6.363)
Allele																				
A	95	9		References	28	76		References	18	86		References	27	77		References	38	66		References
C	119	5			42	82	0.323	1.39 (0.757—2.572)	38	86	0.028	2.111 (1.075—4.242)	53	71	0.012	2.129 (1.169—3.912)	56	68	0.237	1.43 (0.812—2.528)
**VDR BsmI (rs1544410)**																				
Genotype																				
AA	18	1		References	5	14		References	3	16		References	6	13		References	9	10		References
GA	48	4	1	0.667 (0.013—7.393)	14	38	1	1.032 (0.279—4.346)	10	42	1.000	1.27 (0.275—8.069)	13	39	0.786	0.722 (0.202—2.819)	17	35	0.389	0.539 (0.163—1.824)
GG	41	2	1	1.139 (0.018—23.16)	16	27	0.593	1.659 (0.447—6.97)	15	28	0.217	2.857 (0.648 -	21	22	0.325	2.068 (0.588—7.851)	21	22	1	1.061 (0.314—3.609)
Allele																				
A	84	6		References	24	66		References	16	74		References	25	65		References	35	55		References
G	130	8	0.997	1.161 (0.319—3.967)	46	92	0.358	1.375 (0.738—2.596)	40	98	0.075	1.888 (0.945—3.893)	55	83	0.083	1.723 (0.938—3.205)	59	79	0.659	1.174 (0.66—2.096)

Results are expressed as absolute number of patients (percentage). The odds ratio (OR) and 95% confidence intervals (95% CI). CC denotes homozygosity for the C-encoded VDR FokI (rs2228570) polymorphism variant; TT homozygosity for the T-encoded VDR FokI (rs2228570) T polymorphism variant; CT heterozygosity for VDR FokI (rs2228570) polymorphism. CC denotes homozygosity for the C-encoded VDR TaqI (rs731236) polymorphism variant; TT homozygosity for the T-encoded VDR TaqI (rs731236) T polymorphism variant; CT heterozygosity for VDR TaqI (rs731236) polymorphism. AA denotes homozygosity for the A-encoded VDR ApaI (rs7975232) polymorphism variant; CC homozygosity for the C-encoded VDR ApaI (rs7975232) T polymorphism variant; AC heterozygosity for VDR ApaI (rs7975232) polymorphism. AA denotes homozygosity for the A-encoded VDR BsmI (rs1544410) polymorphism variant; GG homozygosity for the G-encoded VDR BsmI (rs1544410) T polymorphism variant; AG heterozygosity for VDR BsmI (rs1544410).

Cytel Studio version 11.1.0 (January 05, 2016).

*VDR* vvitamin D receptor, *BPD* bronchopulmonary dysplasia, *IVH* intraventricular hemorrhage, *NEC* necrotizing enterocolitis, *RDS* respiratory distress syndrome, *ROP* retinopathy of prematurity.

## Discussion

Abnormalities in vitamin D levels have consistently been reported to produce adverse effects on human health and disease development^29^. A physiological response to vitamin D, is regulated by nuclear VDR and its resulting transcription cascade. The overall mechanism of the VDR protein revolves around its binding to the active form of vitamin D, producing a protein interaction with the retinoid X receptor, resulting in the regulation of transcription factors^30^. Such regulation mainly involves the process of mineral metabolism but has also been associated in the regulation of other metabolic pathways pertaining to embryological and immunological responses and development^31^.

The subtle variations in the genetic makeup of VDR, also known as polymorphisms, have been linked to a wide array of diseases including metabolic, immunological, and multiple cancers^32^. Polymorphisms in the VDR gene have also been found to influence neonate development, and is being increasingly explored for its role and prognostic value in certain neonatal complications. Maternal and fetal VDR genotype variants may increase the risk for preterm births^33^. As prematurity carries risk for multiple complications^34^, understanding the role of VDR polymorphisms in such complications become essential for elaborate prognosis. To date, few preterm infant complications have been linked to such polymorphic changes. However, a majority of the preterm complications remain unassessed for their association with VDR polymorphisms.

### Respiratory distress syndrome

RDS is a common complication in preterm children, as a result of underdeveloped lungs, which interferes with the absorption of appropriate levels of oxygen^35^. Glasgow and colleagues argued for the significance of vitamin D playing a role as a prophylactic for respiratory distress in preterm babies^36^. An evaluation of RDS in preterm infants found that high levels of vitamin D decreased the probability of RDS by a ratio of 3.34 times^37^. Both Vitamin D and the VDR receptor influence embryological and perinatal development. VDR TaqI (rs731236) polymorphism, CC and CT genotype has been associated with increased risk of developing RDS in preterm neonates^38^. The authors found that within a TaqI polymorphism, a CC genotype variance was equated with a 5.22 times increased risk in developing RDS, while a TaqI CT genotype variance was 3.26 times more likely present in preterm infants with RDS.

The mechanisms involving the VDR polymorphism with RDS, to date, are not well understood. Within the results of our study, the genetic variability of the VDR gene did not alter the VDR receptor significantly enough in order to influence the incidence of RDS in preterm neonates. A most recent publication by Ustun et al. argue that VDR TaqI polymorphism did influence the incidence of RDS but the Fok1 polymorphism did not^38^. Our study does replicate the lack of Fok1 VDR polymorphism influence on the incidence of RDS but disputes that of the TaqI VDR polymorphism. These antithetical results reveal the importance of further investigation into the influence of VDR polymorphisms on RDS in order to deduce possible genetic risk factors.

### Intraventricular hemorrhage

The pathogenesis of intraventricular hemorrhage, involves a multifactorial etiology, influenced by both environmental and genetic elements^39,40^. Crucial components observed in the pathological variations of IVH include alterations in venous and arterial cerebral blood flow, which have been associated with the fragility of the germinal matrix vasculature^41–43^. To date, the influence of Vitamin D on IVH in preterm infants is up for debate with little to no apparent investigation on its influence of the VDR gene. Elimination of the VDR receptor in knockout mice has revealed alterations in the vascular function of vascular endothelial cells^44^. With the absence of endothelial VDR receptors, Ni and colleagues found a reduction of endothelial nitric oxide synthase (eNOS) expression. Polymorphic changes in endothelial nitric oxide synthase, have been associated with a 3.4-fold increased risk of developing IVH in preterm infants^45^.

According to the results of our study, VDR polymorphisms do not affect the incidence of IVH. The slight variations in the VDR genetic makeup do not appear to influence IVH incidence rates. To the authors' knowledge, no previous study analyzing a potential association between VDR polymorphisms and IVH was identified. It is worth noting, that observations of preterm infants in the German Neonatal Network, showed no detectable differences in incidence frequency of IVH in preterm neonates with high and low Vitamin D levels^46^. Considering the multifactorial nature of IVH, further investigation is required to deduce potential hallmarks of VDR polymorphisms and IVH interactions.

### Bronchopulmonary dysplasia

Both animal and human studies have placed emphasis on the role of vitamin D in proper lung maturation^47,48^. Hypovitaminosis D has been established as a potential risk factor for improper lung regulation and respiratory morbidities, such as RDS and BPD^49^. As such, VDR gene polymorphisms may have slight, but meaningful, variations that mimic vitamin D abnormalities and lead to its consequent effect^32^. In the performed analysis, the VDR gene polymorphism rs7975232 (ApaI) was significantly associated with a higher incidence of BPD. Preterm infants with the receptor genotype CC ApaI (rs7975232) were roughly 4 times more likely to develop BPD, while the infants with the allele C of ApaI (rs7975232) were about twice as likely. This is in contrast to a mirror study performed in 2014 by Koroglu et al.^50^. The study analyzed the same VDR polymorphisms (FokI, BsmI, ApaI, TaqI) in 109 preterm infants (47 of which developed BPD), and concluded that only the FokI receptor polymorphism was associated with an increased frequency of the dysplasia. Such contrasting findings highlight the need for further research concerning VDR polymorphism association to BPD.

### Necrotizing enterocolitis

In the pathogenesis of NEC, the transmembrane protein, toll-like receptor 4 (TLR4), and its cascading effect has been established as a possible key component^51^. Abnormalities within this signalling pathway in the intestines leads to an unregulated activation of nuclear factor-kappa B (NF-κB), that in turn increases the production of proinflammatory cytokines^52,53^. This sequence of events leads to tissue damage and eventual NEC. Vitamin D plays a protective role in the intestines by regulating TLR4, and a deficiency in this vitamin has been reported as a feature of preterm NEC infants^54,55^. Nonetheless, to the authors’ knowledge, no previous studies have explored the potential role of VDR polymorphisms in NEC. The results of our analysis show that preterm infants with the VDR allele C of ApaI (rs7975232) were twice as likely to develop NEC. Such a finding highlights the possible role of VDR genetic variation on the overall interaction between vitamin D and the TLR4 cascade. An increasing number of reports have placed emphasis on the genetic predisposition of preterm infants to NEC^56–61^. However, due to lack of extensive research and coherence of results, validating studies remain necessary to elucidate the relationship between gene variation and NEC^62^. Including VDR polymorphisms in future genetic studies may prove beneficial in understanding certain infants vulnerability to NEC.

### Retinopathy of prematurity

Two phases for ROP pathogenesis are generally recognized. The first phase involves suppressed insulin-like growth factor-1 (IGF-1) and VEGF, due to a hyperoxic neonatal state, that leads to a disruption of retinal vascularization^63^. Eventually, the resulting hypoxic stress from improper vascularization of the retina, stimulates the production of excess angiogenic factors (VEGF, IGF-1) that lead to new abnormal blood vessel formation (second phase)^64^. In a study evaluating the relationship between vitamin D levels and ROP, Kabatas EU et al. found that infants with lower serum 25-hydroxy vitamin D were 1.14 times more likely to develop ROP^65^. They hypothesized that vitamin D deficiency may facilitate angiogenic conditions in the retina, reinforcing the development of ROP. Such a link is yet to be extensively explored.

To the authors' knowledge, this is the first study analyzing a potential association between VDR polymorphism and ROP. The mild variations in the genetic makeup of the receptor did not appear to influence ROP incidence. Polymorphisms, particularly those relating to VEGF, have previously been highlighted as risk factors in ROP, underlining a possible genetic predisposition^66–68^. However, according to the results of our study, VDR polymorphisms remain inconsequential to ROP development, and require further investigation.

In the presented study, the vitamin D levels in the preterm infants were not analysed for a possible correlation to the prematurity complications. The assessment protocols focused solely on the genetic background of these complications. Including such a variable in further analysis can assist in deconstructing the relationship between receptor polymorphisms and development of preterm complications.

Notably, in recent years, alternative pathways of vitamin D activation have also been identified. Slominski AT and colleagues defined a novel pathway for metabolizing vitamin D3, revolving around the mitochondrial enzyme CYP11A1, and found predominantly in the adrenal glands, placenta, and keratinocytes of the epidermis^69–71^. Understanding the role of this novel pathway and its similar, yet distinct, vitamin D hydroxyderivatives (and its receptors) may further unveil some clinical relevance to preterm infants and their associated complications. In addition to varying pathways of vitamin D activation, different receptors for the active metabolite should also be taken into consideration for further analysis^72^. Receptors such as ROR (both α and γ) and AhR have shown interactions with varying vitamin D derivatives, underlining the misconception that VDR is the sole receptor for the vitamin^73–75^. Exploring the downstream influence of ROR and AhR gene polymorphisms, alongside VDR, allows for more elaborate understanding of this pivotal and versatile vitamin.

Despite providing fundamental insight into the relationship between VDR polymorphisms and preterm infant complications, the above-mentioned genetic analysis acts as preliminary and requires further investigation. Larger sample sizes in non-homogenous populations can provide valuable information concerning patterns of pathogenesis and genetic susceptibility of certain infants. Allocating efforts and resources into the role of polymorphisms, including that of VDR, in preterm complications carry promising potential and can allow for future construction of more personalized care.

## Conclusion

Exploring genetic variations in the vitamin D physiological pathway of functioning may broaden the current understanding of genetic susceptibility to preterm complications. The genotype CC of ApaI (rs7975232) VDR gene polymorphism was associated with a 3.8 times increase in likelihood of developing BPD in preterm infants. Furthermore, infants with the allele C of Apa I (rs7975232) were twice as likely to develop BPD and NEC. Including VDR polymorphisms in future genetic assessments of preterm complications is advised.

## References

[1] Kato S (2000). The function of vitamin d receptor in vitamin D action. J. Biochem..

[2] Pike JW, Meyer MB (2010). The vitamin d receptor: new paradigms for the regulation of gene expression by 1,25-dihydroxyvitamin d3. Endocrin. Metab. Clin..

[3] Knabl J (2017). Role of placental vdr expression and function in common late pregnancy disorders. Int. J. Mol. Sci..

[4] Bischoff-Ferrari HA, Borchers M, Gudat F, Durmuller U, Stahelin HB, Dick W (2004). Vitamin d receptor expression in human muscle tissue decreases with age. J. Bone Miner. Res..

[5] Bikle DD (2014). Vitamin d metabolism, mechanism of action, and clinical applications. Chem. Biol..

[6] Taymans SE, Pack S, Pak E (1999). The human vitamin d receptor gene (vdr) is localized to region 12cen-q12 by fluorescent in situ hybridization and radiation hybrid mapping: genetic and physical vdr map. J. Bone Min. Res..

[7] Mukhtar, M. et al. Vitamin d receptor gene polymorphism: an important predictor of arthritis development. *BioMed Res. Int. 6*, ID8326246 (2019). 10.1155/2019/8326246 PMC6442488 31011579

[8] Yu Z-H, Chen M, Zhang Q-Q, Hu X (2018). The association of vitamin d receptor gene polymorphism with lung cancer risk: an update meta-analysis. Comb. Chem. High T. Scr..

[9] Imani D, Razi B, Motallebnezhad M, Rezaei R (2019). Association between vitamin d receptor (vdr) polymorphisms and the risk of multiple sclerosis (ms): an updated meta-analysis. BMC Neurol..

[10] Apaydın M (2019). The vdr gene fokI polymorphism is associated with gestational diabetes mellitus in Turkish women. BMC Med. Genet..

[11] Angel B (2018). The association of vdr polymorphisms and type 2 diabetes in older people living in community in Santiago de Chile. Nutr. Diabetes..

[12] Han J-C, Du J, Zhang Y-J (2016). Vitamin d receptor polymorphisms may contribute to asthma risk. J. Asthma..

[13] Laczmanski L, Milewicz A, Lwow F (2013). Vitamin d receptor gene polymorphism and cardiovascular risk variables in elderly Polish subjects. Gunecol. Endocrinol..

[14] Wu J, Shang D-P, Yang S (2016). Association between the vitamin d receptor gene polymorphism and osteoporosis. Biomed. Rep..

[15] Gelder CM, Hart KW, Williams OM (2000). Vitamin d receptor gene polymorphisms and susceptibility to mycobacterium malmoense pulmonary disease. J. Infect. Dis..

[16] weet, L. R., Keech, C., Klein, N. P. et al. Respiratory distress in the neonate: case definition & guidelines for data collection, analysis, and presentation of maternal immunization safety data. *Vaccine*. **35**, 6506–17 (2017) 10.1016/j.vaccine.2017.01.046 PMC5710987 29150056

[17] Sweet DG, Carnielli V, Greisen G (2019). European consensus guidelines on the management of respiratory distress syndrome: 2019 update. Neonatology..

[18] Papile LA, Burstein J, Burstein R, Koffler H (1978). Incidence and evolution of subependymal and intraventricular hemorrhage: a study of infants with birth weights less than 1,500 gm. J. Pediatr..

[19] Fischer HS (2018). Sustained inflations and avoiding mechanical ventilation to prevent death or bronchopulmonary dysplasia: a meta-analysis. Eur. Respir. Rev..

[20] Nyman A, Korhonen T, Munck P, Parkkola R (2017). Factors affecting the cognitive profile of 11-year-old children born very preterm. Pediatr. Res..

[21] Isayama T, Lee SK, Yang J, Lee D (2017). Revisiting the definition of bronchopulmonary dysplasia effect of changing panoply of respiratory support for preterm neonates. JAMA Pediatr..

[22] Jensen EA, Dysart K, Gantz MG, McDonald S (2019). Eunice Kennedy Shriver National Institute of Child Health and Human Development Neonatal Research Network: the diagnosis of bronchopulmonary dysplasia in very preterm infants: an evidence-based approach. Am. J. Respir. Crit. Care Med..

[23] Jobe AH, Bancalari E (2001). Bronchopulmonary dysplasia. Am. J. Respir. Crit. Care Med..

[24] Gregory KA, DeForge CE, Natale KM (2011). Necrotizing enterocolitis in the premature infant: neonatal nursing assessment, disease pathogenesis, and clinical presentation. Adv. Neonatal Care..

[25] Rich BS, Dolgin SE (2017). Necrotizing enterocolitis. Pediatr. Rev..

[26] Gotz-Wieckowska A (1997). Ranibizumab after laser photocoagulation failure in retinopathy of prematurity (ROP) treatment. Sci. Rep..

[27] The International Classification of Retinopathy of Prematurity Revisited (2005). Arch. Ophthalmol.

[28] Gobel W, Richard G (1993). Retinopathy of prematurity-current diagnosis and management. Eur J. Pediatr..

[29] Zhang R, Naughton DP (2010). Vitamin d in health and disease: current perspectives. Nutr. J..

[30] Barsony J, Prufer K (2002). Vitamin d receptor and retinoid x receptor interactions in motion. Vitam. Horm..

[31] Nair R, Maseeh A (2012). Vitamin d: the “sunshine” vitamin. J. Pharmacol. Pharmacother..

[32] Valdivielso JM, Fernandez E (2006). Vitamin d receptor polymorphisms and diseases. Clin. Chim. Acta..

[33] Barchitta M (2018). Single nucleotide polymorphisms in vitamin d receptor gene affect birth weight and the risk of preterm birth: results from the “mamma & bambino” cohort and a meta-analysis. Nutrients..

[34] Randis TM (2008). Complications associated with premature birth. AMA J. Ethics..

[35] Course C, Chakraborty M (2020). Management of respiratory distress syndrome in preterm infants in wales: a full audit cycle of a quality improvement project. Sci. Rep..

[36] Glasgow JFT, Thomas PS (1977). Rachitic respiratory distress in small preterm infants. Arch. Dis. Child..

[37] Ataseven F, Aygun C, Okuyucu A (2013). Is vitamin D deficiency a risk factor for respiratory distress syndrome?. Int. J Vitam. Nutr. Res..

[38] Ustun N, Eyerci N, Karadag N (2020). Association of vitamin d receptor gene FokI and TaqI polymorphisms and risk of RDS. J. Matern. Fetal. Neonatal. Med..

[39] Ment LR, Aden U, Lin A (2014). Gene-environment interactions in severe intraventricular hemorrhage of preterm neonates. Pediatr. Res..

[40] Szpecht D, Szymankiewicz M, Seremak-Mrozikiewicz A, Gadzinowski J (2015). The role of genetic factors in the pathogenesis of neonatal intraventricular hemorrhage. Folia Neuropathol..

[41] Kenet G, Kuperman AA, Strauss T, Brenner B (2011). Neonatal ivh: mechanisms and management. Thromb Res..

[42] Ballabh P (2010). Intraventricular hemorrhage in premature infants: Mechanism of disease. Pediatr. Res..

[43] Ballabh P (2014). Pathogenesis and Prevention of Intraventricular Hemorrhage. Clin. Perinatol..

[44] Ni W, Watts SW, Ng M (2014). Elimination of vitamin d receptor in vascular endothelial cells alters vascular function. Hypertension.

[45] Szpecht D (2017). Role of endothelial nitric oxide synthase and endothelin-1 polymorphism genes with the pathogenesis of intraventricular hemorrhage in preterm infants. Sci Rep..

[46] Mannhardt C (2020). Genetic predisposition for vitamin d deficiency is not associated with adverse outcome of very low birth weight infants: a cohort study from the german neonatal network. PLoS ONE.

[47] Zosky GR, Berry LJ, Elliot JG (2011). Vitamin d deficiency causes deficits in lung function and alters lung structure. Am. J. Respir. Crit. Care Med..

[48] Park S-H (2019). Association of vitamin d status at birth and respiratory outcomes in preterm infants. Korean J. Pediatr..

[49] Lykkedegn S, Sorensen GL, Beck-Nielsen SS, Christesen HT (2015). The impact of vitamin d on fetal and neonatal lung maturation. A systematic review. Am. J. Physiol. Lung Cell Mol. Physiol..

[50] Koroglu OA, Onay H, Cakmak B (2014). Association of vitamin d receptor gene polymorphisms and bronchopulmonary dysplasia. Pediatr Res..

[51] Tanner SM, Berryhill TF, Ellenburg JL (2015). Pathogenesis of necrotizing enterocolitis. Am. J. Clin. Pathol..

[52] Takeda K, Akira S (2001). Roles of toll-like receptors in innate immune responses. Genes Cells..

[53] Palsson-McDermott EM, O’Neill LAJ (2004). Signal transduction by the lipopolysaccharide receptor, toll-like receptor-4. Immunology.

[54] Shi Y, Liu T, Zhao X (2018). Vitamin d ameliorates neonatal necrotizing enterocolitis via suppressing TLR4 in a murine model. Pediatr. Res..

[55] Yang L-R, Li H, Zhang T, Zhao R-C (2018). Relationship between vitamin d deficiency and necrotizing enterocolitis in preterm infants. Zhongguo Dang Dai Er Ke Za Zhi.

[56] Moonen RM (2020). Risk of necrotizing enterocolitis associated with the single nucleotide polymorphisms VEGF C-2578A, IL-18 C-607A, and IL-4 receptor α-chain A-1902G: a validation study in a prospective multicenter cohort. Front. Pediatr..

[57] Szebeni B, Szekeres R, Rusai K (2006). Genetic polymorphisms of CD14, toll-like receptor 4, and caspase-recruitment domain 15 are not associated with necrotizing enterocolitis in very low birth weight infants. J. Pediatr. Gastr. Nutr..

[58] Szpecht D, Neumann-Klimasinska N, Blaszczynski M (2018). Candidate gene analysis in pathogenesis of surgically and non-surgically treated necrotizing enterocolitis in preterm infants. Mol. Cell. Biochem..

[59] Banyasz I, Bokodi G, Vasarhelyi B (2006). Genetic polymorphisms for vascular endothelial growth factor in perinatal complications. Eur. Cytokine. Netw..

[60] Sankararaman S (2013). The prevalence of platelet activating factor acetylhydrolase single nucleotide polymorphisms in relationship to necrotizing enterocolitis in northwest louisiana infants. Springerplus..

[61] Sampath V, Le M, Lane L (2011). The NFKB1 (g-24519delATTG) variant is associated with necrotizing enterocolitis (NEC) in premature infants. J. Surg. Res..

[62] Cuna A, George L, Sampath V (2018). Genetic predisposition to necrotizing enterocolitis in premature infants: current knowledge, challenges, and future directions. Semin. Fetal. Neonatal. Med..

[63] Saugstad OD (2006). Oxygen and retinopathy of prematurity. J. Perinatol..

[64] Smith LEH (2003). Pathogenesis of retinopathy of prematurity. Semin. Neonatol..

[65] Kabatas EU, Dinlen NF, Zenciroglu A (2017). Relationship between serum 25-hydroxy vitamin D levels and retinopathy of prematurity. Scott. Med. J..

[66] Banyasz I, Bokodi G, Vannay A (2006). Genetic polymorphisms of vascular endothelial growth factor and angiopoietin 2 in retinopathy of prematurity. Curr. Eye Res..

[67] Ali AA, Hussien NF, Samy RM, Husseiny KA (2015). Polymorphisms of vascular endothelial growth factor and retinopathy of prematurity. J. Prediat. Ophth. Strab..

[68] Lei X-J, Zhao Y-X, Qiao T (2018). Influence of polymorphisms in VEGF, ACE, TNF and GST genes on the susceptibility to retinopathy of prematurity among Chinese infants. Int. J. Ophthalmol..

[69] Slominski AT, Kim T-K, Shehabi HZ (2012). In vivo evidence for a novel pathway of vitamin D3 metabolism initiated by P450scc and modified by CYP27B1. FASEB J..

[70] Slominski AT, Li W, Kim T-K (2015). Novel activities of CYP11A1 and their potential physiological significance. J. Steroid Biochem. Mol. Biol..

[71] Slominski AT, Kim T-K, Li W (2015). Detection of novel CYP11A1-derived secosteroids in the human epidermis and serum and pig adrenal gland. Sci. Rep..

[72] Slominski AT, Chaiprasongsuk A, Janjetovic Z (2020). Photoprotective properties of vitamin D and lumisterol hydroxyderivatives. Cell Biochem. Biophys..

[73] Slominski AT, Kim T-K, Hobrath JV (2017). Endogenously produced nonclassical vitamin D hydroxy-metabolites act as “biased” agonists on VDR and inverse agonists on RORα and RORγ. J. Steroid Biochem. Mol. Biol..

[74] Slominski AT, Kim T-K, Takeda Y (2014). RORα and ROR γ are expressed in human skin and serve as receptors for endogenously produced noncalcemic 20-hydroxy- and 20,23-dihydroxyvitamin D. FASEB J..

[75] Slominski AT, Kim T-K, Janjetovic Z (2018). Differential and overlapping effects of 20,23(OH)2D3 and 1,25(OH)2D3 on gene expression in human epidermal keratinocytes: identification of AhR as an alternative receptor for 20,23(OH)2D3. Int. J. Mol. Sci..

